# Efficacy and Safety of *Acmella oleracea* and *Boswellia serrata* Extract as Add-On Therapy for Chronic Low Back Pain: An Observational, Real-World Cohort Study

**DOI:** 10.3390/ph18121903

**Published:** 2025-12-17

**Authors:** Mariateresa Giglio, Consalvo Mattia, Pasquale Sansone, Gabriele Finco, Salvatore Sardo, Michele Sofia, Dario Gaetano, Giuseppe Trivelli, Maria Caterina Pace, Fabio Turco, Vincenzo Desiderio, Alberto Corriero, Fara Fornarelli, Antonella Paladini, Sabatino Maione, Livio Luongo, Filomena Puntillo

**Affiliations:** 1 Department of Interdisciplinary Medicine, ICU and Pain Therapy Section, University of Bari “Aldo Moro”, 70124 Bari, Italy; mariateresa.giglio@uniba.it (M.G.); dottoressa.fornarelli@gmail.com (F.F.); filomena.puntillo@uniba.it (F.P.); 2 Department of Anesthesia, Intensive Care Medicine and Pain Therapy, La Sapienza University, 00161 Rome, Italy; consalvo.mattia@uniroma1.it; 3 Department of Women, Child and General and Specialized Surgery, University of Campania “Luigi Vanvitelli”, 80138 Naples, Italy; pasquale.sansone@unicampania.it (P.S.); dario.gaetano@unicampania.it (D.G.); giuseppe.trivelli@unicampania.it (G.T.); mariacaterina.pace@unicampania.it (M.C.P.); 4 Department of Medical Science and Public Health, University of Cagliari, 09124 Cagliari, Italy; gabriele.finco@unica.it (G.F.); salvatore.sardo@unica.it (S.S.); 5 ASST Rhodense, The Hospital of Garbagnate Milanese, 20024 Milan, Italy; msofia@asst-rhodense.it; 6 Cannabiscientia SA, 6900 Lugano, Switzerland; fabio.turco81@yahoo.it; 7 Department of Experimental Medicine, University of Campania “Luigi Vanvitelli”, 80138 Naples, Italy; vincenzo.desiderio@unicampania.it; 8 Department of MESVA, University of L’Aquila, 67100 L’Aquila, Italy; antonella.paladini@univaq.it; 9 Department of Experimental Medicine, Division of Pharmacology, University of Campania “Luigi Vanvitelli”, 80138 Naples, Italy; sabatino.maione@unicampania.it (S.M.); livio.luongo@unicampania.it (L.L.)

**Keywords:** *Acmella oleracea*, analgesia, *Boswellia serrata*, chronic low back pain, neuropathic pain, nutraceutical, phytotherapy, quality of life

## Abstract

**Introduction:** Chronic low back pain (CLBP) with neuropathic components poses a therapeutic challenge due to the limited efficacy and tolerability of conventional pharmacologic options. Botanical extracts such as *Acmella oleracea* and *Boswellia serrata* have demonstrated anti-inflammatory and analgesic properties. This study aimed to explore the role of a food supplement containing a standard formulation of these extracts as an adjunct to standard care in patients with CLBP. **Methods:** In this prospective, multicenter, observational, real-world, cohort study, 103 patients with CLBP and neuropathic pain received a standardized *A. oleracea* and *B. serrata* extract for 8 weeks as an add-on to ongoing therapy. Neuropathic pain was assessed using the painDETECT (PD-Q) and Neuropathic Pain Symptom Inventory (NPSI). General pain intensity (NRS), disability (ODI), quality of life (SF-12), concomitant analgesic use, and safety were also monitored at baseline, and at Weeks 2, 4, and 8. **Results:** PD-Q scores significantly decreased by 13.4% at Week 2, 25.5% at Week 4, and 37.1% at Week 8 and NPSI scores decreased by 15.8%, 24.4%, and 36.9%, respectively (all *p* < 0.0001 vs. baseline). NRS pain intensity improved by 28.0% by Week 8 (*p* < 0.0001). ODI scores reduced by 20.8% (*p* < 0.0001) and SF-12 scores improved by 4.1% (*p* < 0.001) compared to baseline. Use of NSAIDs and gabapentinoids decreased by 23.7%, and 22.2%, respectively (*p* < 0.05). No serious adverse events occurred; mild and transient effects were reported in 8.7% of patients. **Conclusions:** The *A. oleracea* and *B. serrata* extract as adjunctive therapy resulted in significant improvements in neuropathic pain, functional disability, and reduced medication use, with good tolerability. While these findings suggest a potential role for this botanical combination in managing CLBP with neuropathic components, the absence of a control group limits causal inference. Randomized controlled trials are needed to establish efficacy and confirm these preliminary observations.

## 1. Introduction

Low back pain is a leading cause of disability worldwide, affecting approximately 619 million people globally in 2020, with projections indicating that this number could rise to 843 million by 2050 [1]. Although low back pain is often self-limiting, a significant proportion of patients develop chronic low back pain (CLBP), defined as pain persisting for more than 12 weeks, which is frequently accompanied by neuropathic features and central sensitization [2,3]. The presence of a neuropathic component in CLBP is increasingly recognized and supported by epidemiological data. A 2017 meta-analysis estimated that up to 55% of individuals with CLBP exhibit clinically relevant neuropathic pain characteristics [4]. This finding is further supported by large-scale reviews and clinical trials highlighting the coexistence of nociceptive and neuropathic mechanisms in CLBP, often driven by nerve root compression, inflammatory mediator release, and neuroplastic changes in the spinal cord and brain [5,6]. Central sensitization is a maladaptive state in which altered pain processing amplifies pain perception beyond nociceptive input [7,8,9]. This dysregulation results in impaired conditioned pain modulation, meaning a diminished ability of endogenous inhibitory pathways to suppress pain, and enhanced temporal summation of pain, indicating an exaggerated response to repetitive noxious stimuli [2,10]. These neurophysiological changes suggest that CLBP is not merely a musculoskeletal disorder but a dysfunction in pain regulatory pathways, necessitating treatment approaches that address both peripheral inflammation and central pain dysregulation.

Current pharmacological treatments provide inconsistent long-term efficacy and are associated with significant side effects [11]. Neuromodulators (e.g., gabapentinoids and antidepressants), which modulate central pain pathways, often provide only modest symptom relief and, in some patients, are frequently limited by sedation, dizziness, and cognitive impairment [12]. Non-steroidal anti-inflammatory drugs (NSAIDs), though effective for inflammation, offer limited relief in CLBP with neuropathic components and may be associated with gastrointestinal, cardiovascular, and renal complications with prolonged use [12]. Opioids, despite their widespread use, lack evidence for long-term effectiveness in CLBP and pose serious risks, including tolerance, dependence, addiction, and misuse, prompting recommendations for restricted use [11,13]. Given these limitations, there is increasing interest in non-pharmacological approaches or integrative medicine that target both peripheral and central pain mechanisms while mitigating the risks associated with long-term analgesic use.

Botanical compounds with analgesic and anti-inflammatory properties, such as *Acmella oleracea* and *Boswellia serrata*, have shown promise in addressing both peripheral and central sensitization. *Acmella* contains alkylamides, particularly spilanthol, which modulate transient receptor potential (TRP) channels and, indirectly, the endocannabinoid system, both critical in central pain processing and endogenous pain modulation [14]. Preclinical studies suggest that *A. oleracea* reduces peripheral and spinal pain transmission, reinforcing its potential in chronic pain states involving central sensitization [15]. Preclinical data support the role of spilanthol (from *Acmella*) and boswellic acids (from *Boswellia*) in modulating TRP channels, cytokines, and neuroimmune mechanisms [16,17]. In particular, the combination of *A. oleracea* and *B. serrata* may act synergistically to reduce mechanical allodynia, spinal inflammation, and hyperexcitability [14].

*B. serrata* widely known for its anti-inflammatory effects, contains boswellic acids, particularly acetyl keto-beta-boswellic acid (AKBA), which inhibits 5-lipoxygenase, a key enzyme in leukotriene-mediated inflammation [18,19,20]. Beyond its role in peripheral inflammation, *Boswellia* modulates microglial activation and pro-inflammatory cytokines in the central nervous system, mechanisms implicated in neuropathic pain persistence, also through the inhibition of the 15-lipoxygenase [21,22]. Emerging evidence suggests that a fixed-dose combination of *A. oleracea* and *B. serrata* may have synergistic effects in chronic pain modulation. Preclinical studies in neuropathic pain models have demonstrated that this combination significantly reduces mechanical allodynia, spinal neuroinflammation, and neuronal hyperexcitability, reinforcing its potential to attenuate both peripheral and central pain mechanisms [14].

Given the role of neuroimmune dysregulation in CLBP pathophysiology, *A. oleracea* and *B. serrata* may serve as effective adjuncts to standard pharmacological treatments, offering a broader mechanistic effect on chronic pain modulation and potentially reducing reliance on conventional analgesics while mitigating their long-term side effects. The aim of this study was to evaluate the safety and efficacy of a food supplement containing *A. oleracea* and *B. serrata* as an adjunct to standard pain management in patients with CLBP and neuropathic pain features.

## 2. Results

### 2.1. Patients

**Figure 1:**
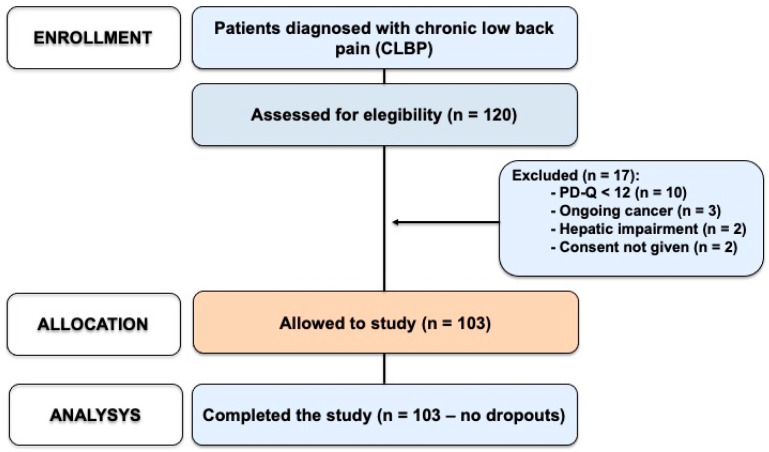
Participant flow diagram for the observational study. A total of 120 patients with a diagnosis of chronic low back pain (CLBP) were screened for eligibility. Seventeen patients were excluded due to: a PD-Q score < 12 (n = 10), ongoing oncologic disease (n = 3), hepatic impairment (n = 2), or refusal to consent (n = 2). The remaining 103 patients were enrolled, allocated to the study, and completed the follow-up without dropouts.

A total of 120 patients were screened for eligibility, of whom 103 met the inclusion criteria and were enrolled in the study (Figure 1). All enrolled patients completed the study without any withdrawals. The mean age of participants was 63.1 (±14.3) years, and the mean body-mass index (BMI) was 26.4 (±4.2) kg/m^2^. Of the enrolled patients, 57 (55.3%) were female and 46 (44.7%) were male. All patients had a confirmed diagnosis of CLBP with a neuropathic component with a PD-Q score ≥ 12 at baseline.

The most common comorbidity was hypertension, present in 24 patients (23.3%), followed by diabetes mellitus (n = 8; 7.8%) and osteoporosis (n = 7, 6.8%). Both chronic obstructive pulmonary disease (COPD) and hypercholesterolemia were reported in 5 patients each (4.9%). Baseline demographic and clinical characteristics of the study population are summarized in Table 1.

At baseline, patients were receiving a range of pharmacological treatments for CLBP (Table 2). NSAIDs (ibuprofen, ketoprofen, diclofenac, nimesulide) were the most frequently used drug class, prescribed to 39 patients, followed by opioids (tapentadol, buprenorphine, tramadol, oxycodone/naloxone, fentanyl) prescribed to 30 patients, and neuromodulators (gabapentin, pregabalin, acetyl-L-carnitine) used by 27 patients. Additionally, 22 patients used paracetamol-based analgesics. A smaller subset of patients (5) used muscle relaxants (thiocolchicoside, tizanidine), and 2 patients used cannabis-based medications. Notably, 8 patients were not receiving any pharmacological treatment at study entry.

The table summarizes the classes of medications used at study entry. A number of patients were receiving more than one pharmacological treatment concurrently. NSAIDs: Non-steroidal anti-inflammatory drugs.

### 2.2. Neuropathic Pain Assessment

**Figure 2:**
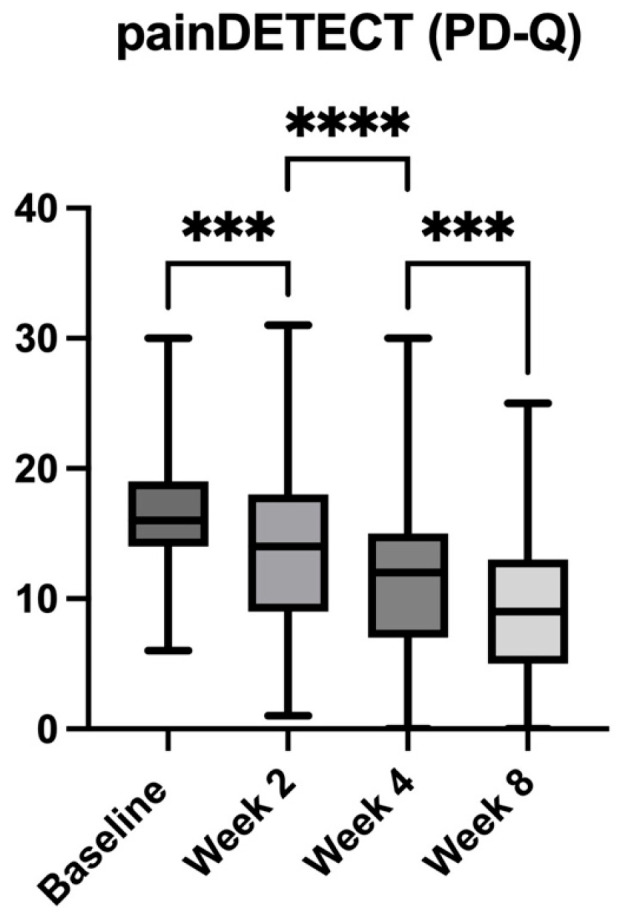
Changes in painDETECT scores over the 8-week study period in patients with chronic low back pain receiving *Acmella oleracea* and *Boswellia serrata* extract as an add-on to standard care. Bars represent mean with range (minimum to maximum values) at baseline, Week 2, Week 4, and Week 8. A significant reduction in neuropathic pain symptoms was observed from baseline to Week 2, Week 4, and Week 8. **** *p* < 0.0001, *** *p* < 0.001.

**Figure 3:**
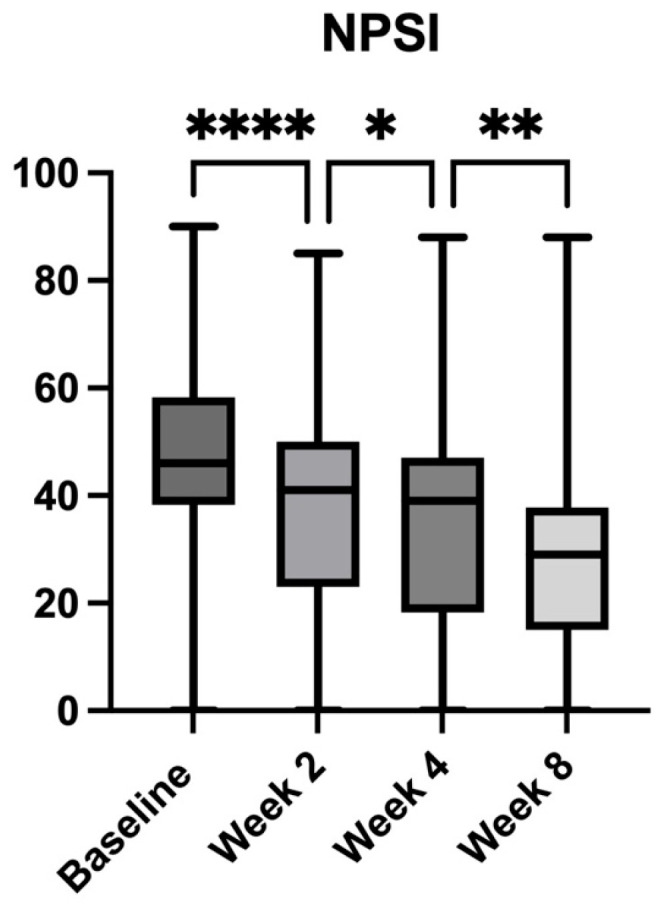
Changes in Neuropathic Pain Symptom Inventory (NPSI) scores over the 8-week study period in patients with chronic low back pain receiving *Acmella oleracea* and *Boswellia serrata* extract as an add-on to standard care. Bars represent mean values with range (minimum to maximum). A progressive and statistically significant reduction in neuropathic pain symptom severity was observed from baseline to Week 2, Week 4, and Week 8. **** *p* < 0.0001, ** *p* < 0.01, * *p* <0.05.

At baseline, all enrolled patients exhibited clinically relevant neuropathic pain, as demonstrated by their PD-Q and NPSI scores. The mean PD-Q score was 16.87 (±4.72) (Figure 2), consistent with a probable neuropathic pain component, while the mean NPSI score was 45.39 (±17.41) (Figure 3), further supporting the presence of neuropathic features in this population. Following the introduction of the *A. oleracea* and *B. serrata* extract as an adjunct to standard analgesic therapy, both PD-Q and NPSI scores showed a statistically significant and clinically meaningful reduction over time.

By Week 2, the PD-Q score had decreased to 13.65 (±6.06), corresponding to a 19.08% reduction compared to baseline (*p* < 0.001) (Figure 2). This downward trend continued through Week 4, with a mean score of 11.76 (±6.65), reflecting a 30.29% reduction (*p* < 0.0001), and reached 9.93 (±6.61) by Week 8, representing an overall 41.14% decrease from baseline (*p* < 0.0001) (Figure 2). When evaluating changes between consecutive follow-ups, a significant reduction was observed from Week 2 to Week 4 (13.85%; *p* < 0.001) and from Week 4 to Week 8 (15.50%; *p* < 0.001). These data confirm that the majority of the improvement occurred within the first month of treatment, with additional clinically significant reductions maintained through Week 8.

In parallel, NPSI scores exhibited a similar consistent downward trend, reinforcing the attenuation of neuropathic pain symptoms over time (Figure 3). The mean baseline NPSI score was 45.39 (±17.41), which decreased to 38.20 (±18.42, *p* < 0.0001 vs. baseline) at Week 2, corresponding to a 15.84% reduction. By Week 4, the mean score had decreased to 34.32 (±19.80, *p* < 0.0001 vs. baseline), reflecting a 24.39% reduction. The greatest improvement was observed at Week 8, with the score further declining to 28.66 (±18.01, *p* < 0.0001 vs. baseline), corresponding to a total reduction of 36.86%. Pairwise comparisons between follow-up visits demonstrated a 10.2% decrease from Week 2 to Week 4 (*p* = 0.0465) and a 16.5% reduction from Week 4 to Week 8 (*p* = 0.002).

Statistically significant improvements were also observed early (Week 2) in several NPSI sub-scores, including squeezing, electric shock-like pain, pins and needles, tingling, and pain evoked by brushing, across follow-up visits (see Table 3). Additionally, pressure sensation showed significant reductions from Week 4 onward, while burning pain and pain evoked by pressure reached significance only at Week 8. Otherwise, pain evoked by cold stimuli was not frequently reported and therefore no significant changes were found.

### 2.3. General Pain Assessment

**Figure 4:**
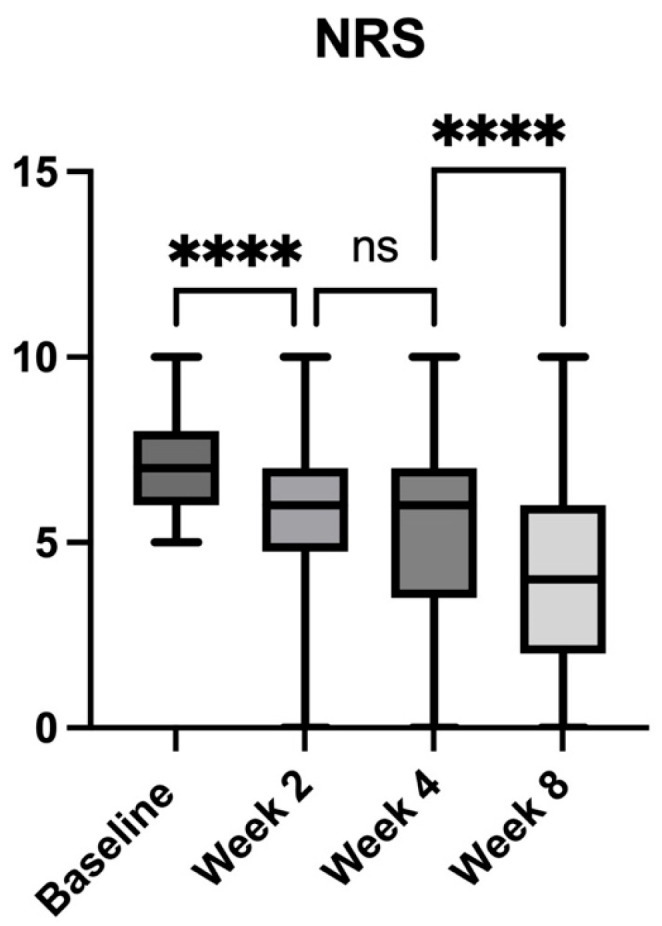
Changes in general pain intensity, measured with the Numeric Rating Scale (NRS), over the 8-week study period in patients with chronic low back pain receiving *Acmella oleracea* and *Boswellia serrata* extract as an add-on to standard care. Bars represent mean values with range (minimum to maximum). A significant and progressive reduction in pain intensity was observed at each follow-up, with the most pronounced improvement by Week 8. **** *p* < 0.0001, ns = not significant.

General pain intensity, assessed using the NRS, showed a significant and progressive reduction following the treatment with the extract (Figure 4). At baseline, the mean NRS score was 7.11 (±1.28), indicating high pain intensity among participants. By Week 2, the score declined to 5.39 (±2.4, *p* < 0.0001 vs. baseline), representing a 24.19% reduction. At Week 4, a further decrease was observed, with a mean score of 5.02 (±2.5, *p* < 0.0001 vs. baseline), corresponding to a 29.39% reduction from baseline. The most substantial improvement occurred at Week 8, where the NRS score decreased to 3.83 (±2.7, *p* < 0.0001 vs. baseline), reflecting a 46.12% reduction in pain intensity compared to baseline.

Between consecutive follow-up visits, pain scores continued to improve. The reduction between Week 2 and Week 4 (6.90%) was not significant (*p* = 0.3369), while the decline between Week 4 and Week 8 was 23.77% (*p* < 0.0001), reaching statistical significance.

### 2.4. Quality of Life Assessment

**Figure 5:**
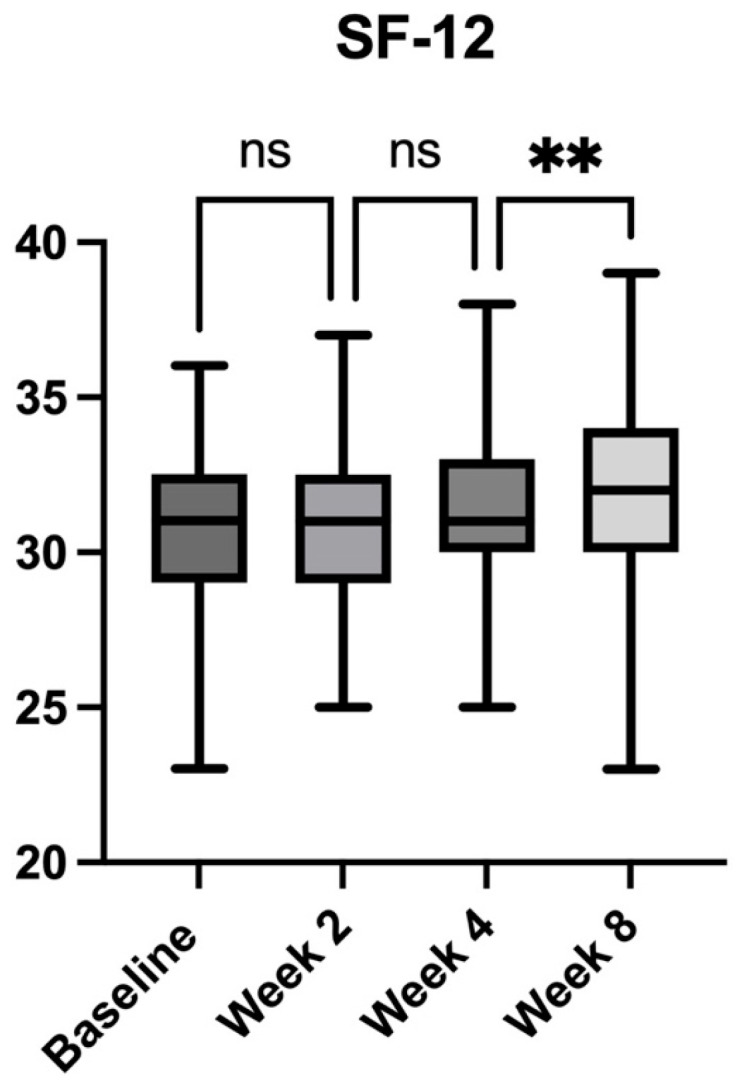
Changes in health-related quality of life (SF-12 scores) over the 8-week study period in patients with chronic low back pain receiving *Acmella oleracea* and *Boswellia serrata* extract as an add-on to standard care. Bars represent mean values with range (minimum to maximum). A significant improvement in SF-12 score was observed at Week 8 compared to baseline. ** *p* < 0.01, ns = not significant.

Health-related quality of life, as measured by the SF-12 questionnaire, demonstrated a modest yet significant improvement over the study period (Figure 5). At baseline, the mean SF-12 score was 30.76 (±2.51), indicating a diminished quality of life associated with CLBP. No significant change was observed at Week 2 (30.74 ± 2.54, *p* = 0.9997 vs. baseline) or Week 4 (31.01 ± 2.48, *p* = 0.8177 vs. baseline). However, by Week 8, a significant improvement emerged, with the mean SF-12 score increasing to 32.02 (±2.94, *p* = 0.0007 vs. baseline), reflecting a 4.1% improvement. Further analysis revealed that the difference between Week 2 and Week 4 was not statistically significant (*p* = 0.5376), but a significant increase occurred from Week 4 to Week 8 (*p* = 0.0098, +3.3%). The most substantial improvement was observed from baseline to Week 8, with a 4.1% increase in SF-12 score (*p* < 0.0001), suggesting a delayed but positive impact of the botanical extract intervention on overall well-being.

**Figure 6:**
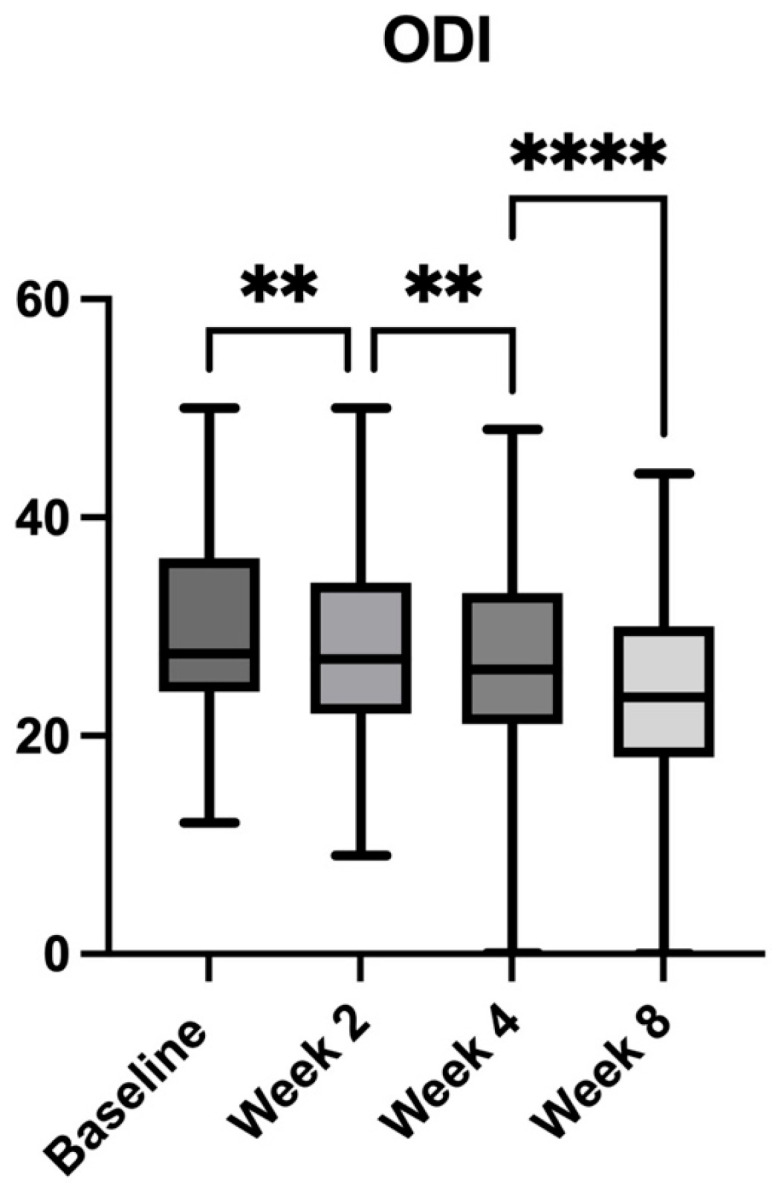
Changes in disability levels assessed by the Oswestry Disability Index (ODI) over the 8-week study period in patients with chronic low back pain receiving *Acmella oleracea* and *Boswellia serrata* extract as an add-on to standard care. Bars represent mean values with minimum and maximum ranges. Statistically significant reductions in disability were observed at all timepoints compared to baseline. **** *p* < 0.0001, ** *p* < 0.01.

Similarly, functional disability, as assessed by the ODI, showed progressive improvement throughout the study (Figure 6). At baseline, the mean ODI score was 29.89 (±8.39). By Week 2, the score declined to 27.94 (±8.12, *p* = 0.004 vs. baseline), corresponding to a 6.53% reduction. At Week 4, the ODI score further decreased to 26.33 (±8.80, *p* < 0.0001 vs. baseline), representing an 11.91% improvement. The most significant improvement was noted at Week 8, where the mean ODI score reached 23.68 (±8.33, *p* < 0.0001 vs. baseline), corresponding to a 20.77% reduction in disability. Additional analyses of consecutive time points showed that the decrease from Week 2 to Week 4 was 5.81% (*p* = 0.0095), and from Week 4 to Week 8, the reduction was 10.13% (*p* < 0.0001). These findings highlight a clinically significant enhancement in functional capacity and quality of life, after the adjunct of the botanical extract to the standard analgesic therapy.

#### Change in Concomitant Medication Use

Analgesic medications consumption was mildly reduced during the study, indicating a lowered reliance on pharmacological pain management following treatment with the *A. oleracea* and *B. serrata* extract (Table 4). The most notable reduction was observed in on demand NSAID use, which decreased from 39 patients at baseline to 30 by Week 8, corresponding to a 23.1% reduction (*p* < 0.05). Gabapentinoids use also significantly declined, from 27 to 21 patients, representing a 22.2% reduction (*p* < 0.05).

In contrast, opioid use showed only a modest reduction (from 30 to 28 patients, 6.7%) and did not reach statistical significance. No significant changes were observed in paracetamol, muscle relaxant, or cannabis use, which remained stable over time. The number of patients requiring no pharmacological treatment increased from 8 at baseline to 12 at Week 8; however, this trend was not statistically significant.

This table presents the number of patients using each class of medication at Baseline, Week 2, Week 4, and Week 8. Percentage reductions and *p*-values refer to the comparison between Week 8 and Baseline. Statistical significance was assessed using the chi-square test of independence or Fisher’s exact test, as appropriate. Fisher’s exact test was applied when expected cell counts were <5. A *p*-value < 0.05 was considered statistically significant. The “No treatment” category represents patients who did not require any pharmacological pain management. % Reduction and *p* values are all Week 8 compared to baseline. n.s = non-significant.

### 2.5. Safety and Tolerability

The *A. oleracea* and *B. serrata* extract was well tolerated throughout the study period, with no serious adverse events reported (Table 5). Among the 103 enrolled patients, mild and transient adverse effects were observed in 9 patients (8.7%), with the most reported events being mild gastrointestinal discomfort (n = 4) and transient dizziness (n = 3). Two patients reported mild skin reactions, which resolved spontaneously without the need for intervention.

No patient discontinued treatment due to adverse effects, and no clinically significant abnormalities in vital signs or laboratory parameters were observed. Additionally, no allergic reactions were observed, and no cases of treatment-related hepatic or renal dysfunction were reported. The overall safety profile suggests that the extract was well tolerated and did not contribute to any significant adverse outcomes, further supporting its use as a complementary approach for managing chronic low back pain with a neuropathic component.

The table summarizes the adverse events reported throughout the study. No serious adverse events or treatment-related discontinuations occurred in all 103 patients analyzed. Mild and transient adverse effects were observed in 8.7% of patients, with the most frequent being mild gastrointestinal discomfort (3.9%) and transient dizziness (2.9%). No significant abnormalities in laboratory parameters or vital signs were reported.

## 3. Discussion

CLBP with neuropathic components remains a challenging condition, frequently inadequately controlled by standard pharmacological approaches. In this context, adjunctive strategies targeting both nociceptive and neuropathic mechanisms are increasingly explored. This real world study evaluated the clinical analgesic impact of a standardized combination of *A. oleracea* and *B. serrata* administered as add on therapy in patients with neuropathic CLBP.

The significant reduction in PD-Q and NPSI scores by Week 4, which was the study’s primary endpoint, indicates a clinically significant attenuation of neuropathic pain symptoms. Notably, this analgesic effect was sustained and even enhanced through Week 8, suggesting progressive neuroplastic changes that extend beyond an acute anti-inflammatory response. The statistically significant improvement observed in several NPSI sub-scores, including squeezing, electric shock-like pain, pins and needles, tingling, and pain evoked by brushing, may reflect the extract’s modulatory action on the multiple overlapping mechanisms involved in radicular pain, including nociceptive, inflammatory, and neuropathic pathways, which collectively contribute to a mixed radicular pain state [23].

Several mechanisms may underlie the observed analgesic effects. *B. serrata* is well-documented for its anti-inflammatory properties, primarily through inhibition of 5- and 15-lipoxygenase, thereby reducing inflammatory mediators involved in neuroinflammation, a key driver of neuropathic pain [18,24,25,26]. Moreover, the ability of boswellic acids to modulate cytokine release, including TNF-α and IL-1β, suggests an additional mechanism by which the extract could mitigate central sensitization and hyperexcitability of spinal neurons [27,28]. A randomized, double-blind, placebo-controlled trial highlighted the role of *B. serrata* in chronic pain management, including CBLP, showing a significantly reduced pain intensity and improved functional outcomes [29]. Additionally, Boswellia has been shown to possess direct analgesic properties, as evidenced by its efficacy in reducing experimentally induced mechanical pain in healthy volunteers, supporting its role in modulating peripheral pain sensitivity [30]. Furthermore, in patients with osteoarthritis of the knee, a *B. serrata* extract provided clinically significant pain relief and improved joint function, reinforcing its broader therapeutic potential in inflammatory and degenerative pain conditions [31].

*A. oleracea*, by contrast, contains bioactive alkylamides such as spilanthol, which have shown analgesic and anti-inflammatory effects. Though its full pharmacodynamics are not yet fully understood, spilanthol appears to modulate Transient Receptor Potential (TRP) channels, particularly TRPV1, which is central to pain signaling [17,32]. Preclinical work supports its antinociceptive role through inhibition of neurotransmitter release and reduction in cytokine expression [33]. The rapid onset of pain relief observed in the present study, with significant reductions in PD-Q and NPSI scores by Week 2, aligns with these proposed mechanisms and support the hypothesis that the extract exerts its effects through both immediate anti-inflammatory actions and longer-term neuroplastic adaptations. Fusco and colleagues provided additional insights into the mechanisms by which this extract combination may modulate pain processing [20]. Their preclinical study demonstrated that treatment with *A. oleracea* and *B. serrata* resulted in reduced neuronal overexcitation and spinal microgliosis in a model of inflammatory pain. The observed reduction in microglial activation and pro-inflammatory cytokine release aligns with the reductions in neuropathic pain scores seen in our study and highlights the potential for broader applications of this phytotherapeutic combination in other chronic pain conditions [34]. Moreover, the isobolographic approach adopted in the study by Boccella et al. further substantiates a synergistic effect of this extract combination [34].

Beyond neuropathic pain modulation, the observed improvements in general pain intensity, as assessed by the NRS, provide further support for the analgesic efficacy of the extract, when added to the standard therapy for CLBP. The reduction in NRS scores by 13.1% at Week 2, 19.2% at Week 4, and 28.0% at Week 8 indicates that the intervention was effective across different pain mechanisms. Again, given the complex nature of CLBP, which includes both nociceptive neuropathic and mixed components, these findings suggest that the extract may exert a broader spectrum of analgesic activity beyond strictly neuropathic mechanisms [35]. The progressive reduction in NRS scores over time also aligns with the hypothesis that longer-term use of the extract may facilitate neuroadaptive processes that contribute to sustained pain relief.

Importantly, the analgesic benefits of the extract translated into significant improvements in health-related quality of life and functional disability, as demonstrated by SF-12 and ODI scores. While significant pain relief was evident by Week 4, improvements in disability and quality of life became more pronounced by Week 8. This suggests that while pain reduction is an early and direct outcome, functional recovery requires sustained analgesia to facilitate improvements in mobility, daily activities, and psychological well-being. Given the bidirectional relationship between pain perception and physical disability, it is plausible that reductions in pain hypersensitivity and central sensitization contributed to an improved capacity for movement, ultimately leading to better disability outcomes.

When used as an adjunct to standard analgesic therapy, the botanical extract appeared to contribute to additional improvements in pain control. Standard treatments for CLBP, particularly in cases with neuropathic features, often show limited effectiveness. Indeed, NSAIDs provide only modest short-term relief in CLBP and have limited efficacy in neuropathic pain, as they primarily target peripheral inflammation rather than central sensitization [36,37]. Neuromodulators can reduce pain by 20–30% but are often poorly tolerated due to sedation, dizziness, and cognitive side effects [38,39]. Opioids have limited long-term utility and may lead to tolerance, dependence, and opioid-induced hyperalgesia [40]. In this context, the addition of the extract was associated with a progressive reduction in pain scores, including a 24.6% decrease in PD-Q at Week 4 and 36.2% by Week 8, as well as significant improvements in NRS and NPSI scores. These clinical benefits were paralleled by a significant reduction in the use of concomitant medications, particularly NSAIDs (23.1% reduction, *p* < 0.05) and gabapentinoids (22.2% reduction, *p* < 0.05). While opioid use declined slightly (6.7%), this change did not reach statistical significance. No significant variations were observed in the use of paracetamol, muscle relaxants, or cannabis. These findings highlight the therapeutic potential of the *A. oleracea* and *B. serrata* extract as a potential adjunctive intervention for the management of CLBP with neuropathic features. This may be particularly relevant for patients who exhibit intolerance to, or insufficient response from, conventional pharmacological treatments. Moreover, the supplement was well tolerated, with no serious adverse events reported. Mild transient side effects occurred in fewer than 9% of patients. This is consistent with prior literature on Boswellia and Acmella, which both exhibit high safety profiles in nutraceutical formulations.

By concurrently attenuating pain intensity and reducing reliance on standard analgesic medications, the extract may represent a safer and more integrative strategy for sustained pain control in this patient population.

Despite these promising findings, several limitations should be acknowledged. The lack of a placebo-controlled design precludes definitive conclusions regarding causality, and the observed improvements could partially reflect natural symptom fluctuations or a placebo response. However, the magnitude and consistency of pain reduction across multiple validated scales argue against a purely placebo-driven effect, particularly considering that the enrolled patients were experiencing severe, treatment-refractory pain prior to receiving the extract, making a substantial placebo response unlikely in this context. Importantly, the decision not to include a control or placebo group was driven by ethical and practical considerations. All participants had already undergone at least three months of standard analgesic therapy without adequate relief, and withholding further active intervention by randomizing them to a placebo was deemed ethically inappropriate in a population with persistent and disabling pain. As such, the study was designed to reflect a real-world clinical scenario in which an add-on treatment is offered in response to insufficient therapeutic outcomes.

Furthermore, the primary objective of the study was exploratory and hypothesis-generating. Observational cohorts such as this are common in early-phase nutraceutical research to assess safety and tolerability, and to detect signals, before conducting resource-intensive randomized controlled trials [41].

The use of multiple, converging outcome measures, such as pain intensity, neuropathic pain indices, and functional assessments, provided internal consistency and strengthened the interpretability of the findings despite the lack of a comparator group. Moreover, introducing a placebo control group would have raised ethical concerns, since all participants were experiencing persistent pain despite standard therapy. Offering an add-on intervention while randomizing a subset of patients to receive no additional treatment was considered inappropriate in this symptomatic and treatment-refractory population.

Additionally, while the 8-week follow-up period was sufficient to capture early efficacy signals, longer studies are needed to determine whether the analgesic effects are sustained over extended periods and whether further reductions in medication use can be achieved.

Finally, the study population was limited to patients with CLBP, and while these findings may be generalizable to other pain conditions, further research is necessary to confirm efficacy in broader chronic pain populations.

## 4. Material and Methods

### 4.1. Study Design

This is an open-label, multicenter, prospective, observational, real-world, cohort study conducted to assess the safety and clinical utility of a food supplement containing *A. oleracea* and *B. serrata* extracts (Nervana^®^, Sanitas Farmaceutici, Milan, Italy) when added to conventional pharmacological therapy in patients with CLBP of neuropathic characteristics. Importantly, the supplement was introduced only after a minimum of 3 months of standard treatment (including NSAIDs, neuromodulators, and/or other analgesics) had failed to provide adequate symptom relief, as judged by the treating physician. The study was conducted at four hospital centers in Italy between January and September 2024, in accordance with Good Clinical Practice and the Declaration of Helsinki (1975, as revised in 2008). It adhered to STROBE guidelines for observational research, using standardized eligibility criteria, validated outcome measures, and structured data collection to minimize selection, measurement, and reporting bias [42]. The aim was to evaluate the contribution of the botanical formulation to symptom improvement and medication reduction in a naturalistic setting, rather than isolate its effects under tightly controlled experimental conditions. Thus, the intervention reflects a pragmatic, add-on strategy in a population with documented suboptimal response to existing therapies. Given this context, a placebo group was not included, as the likelihood that the observed improvements were due to a placebo effect is considered low and, moreover, the study was specifically designed to mirror real-world clinical practice and evaluate the supplement’s contribution as part of a multidrug regimen, rather than to isolate its mechanism of action under experimental conditions. Ethical approval was obtained from the Ethical Committee of Policlinico Hospital of Bari (CIP-04-22, 13 October 2022), and all patients provided written informed consent prior to enrollment.

### 4.2. Study Population

The study population consisted of adult patients diagnosed with CLBP with a neuropathic component who had experienced an insufficient response to at least 3 months of conventional pharmacological therapy. Eligible individuals were outpatients consecutively enrolled on a voluntary basis, from the pain management clinics of the participating centers and selected based on the following standardized inclusion and exclusion criteria to ensure homogeneity and clinical relevance [43].

*Inclusion Criteria:*

- Chronic low back pain persisting for more than 3 months.
- Numeric Rating Scale (NRS) pain score > 4 at baseline.
- Neuropathic pain as supported by clinical evaluation.
- PAIN DETECT (PD-Q) score > 12.

*Exclusion Criteria:*

- Pregnancy or lactation.
- Pain caused by any other documented neuropathy.
- Use of any other dietary supplements.
- Age < 18 years.
- Chronic renal insufficiency (stage > III).
- Severe hepatic impairment (CHILD B or C).
- Active oncological disease.
- Inability to provide informed consent.

### 4.3. Study Procedures

- Screening Visit: Assessment of eligibility according to the inclusion criteria, medical history.
- Baseline Visit: Enrollment of eligible participants, evaluation of pain scores with NRS, PD-Q and neuropathic pain symptom inventory (NPSI), function with Oswestry disability index (ODI) and quality of life with Short Form Health Survey (SF-12). The food supplement treatment started.
- At each follow up visit (week 2, week 4 and week 8): the same questionnaires were collected with treatment adherence and adverse events. The clinical practice was not modified by any protocol instruction as per the observational design of the study. Therefore, analgesics and neuromodulators could be modified accordingly by the treating physician; any changes were registered together with rescue therapy.

To monitor compliance, participants were provided with an infographic with dosing instructions and a tracking calendar, where they marked daily supplement intake. These records were reviewed at each visit.

### 4.4. Data Collection

At the screening visit, the investigators obtained a detailed medical history, including the following:

- Approximate date of pain onset, as recalled by the patient.
- Current pain treatment regimen, including prescribed and over-the-counter medications.
- Comorbid conditions, previous and current medical history.
- Medication history, including chronic use of any prescribed drugs or dietary supplements.
- A comprehensive physical examination was conducted at baseline, which included height and weight measurements.

### 4.5. Questionnaire Administration

Questionnaires were administered in a quiet and consistent environment at each visit. To minimize bias, participants were not informed of any diagnostic test results before completing the questionnaires. No time constraints were imposed, and patients were encouraged to provide accurate and comprehensive responses. Investigators did not assist or interpret responses, but patients were allowed to re-read instructions if clarification was needed. All questionnaires were recorded at baseline, Week 2, Week 4, and Week 8.

#### 4.5.1. Pain DETECT Questionnaire

The PD-Q was used to evaluate the presence and intensity of neuropathic pain components in CLBP patients [44]. The questionnaire consisted of nine items, assessing pain characteristics, intensity, and distribution. A score of >12 indicated a probable neuropathic component, while a score of <12 suggested primarily nociceptive pain. The PD-Q score was collected at baseline and Week 4 to assess changes in neuropathic pain symptoms following the intervention. A reduction in PD-Q score indicate a decrease in neuropathic pain features, potentially reflecting improvements in pain modulation and central sensitization mechanisms.

#### 4.5.2. Neuropathic Pain Symptom Inventory

This validated questionnaire consists of 10 items, evaluating different dimensions of neuropathic pain, including superficial spontaneous pain (burning), deep spontaneous pain (pressing, squeezing), paroxysmal pain (electric shocks, stabbing), evoked pain (evoked by brushing, pressure, cold stimuli), and paresthesia/dysesthesia (pins and needles, tingling).

Each item was rated on a numerical scale (0–10), with higher scores indicating greater neuropathic pain intensity. The total NPSI score was calculated by summing individual item scores, with a maximum possible score of 100.

#### 4.5.3. Numeric Rating Scale

Pain intensity was measured using the numeric rating scale (NRS), a 0 to 10 scale where 0 represented no pain, and 10 indicated the worst imaginable pain [45]. Patients rated their average pain intensity over the preceding 24 h.

#### 4.5.4. Quality of Life

The Short Form Health Survey (SF-12) was administered to evaluate the impact of chronic pain on overall quality of life [46]. This validated instrument measured physical and mental health domains, generating a Physical Component Summary (PCS) score and a Mental Component Summary (MCS) score. Higher SF-12 scores indicated better quality of life.

#### 4.5.5. Oswestry Disability Index

Functional impairment due to low back pain was assessed using the Oswestry disability index (ODI), a 10-item questionnaire evaluating daily activity limitations (e.g., mobility, self-care, lifting, standing, and social life) [47]. ODI scores ranged from 0 to 100, with higher scores indicating greater disability.

### 4.6. Intervention

Prior to the introduction of the food supplement, patients had been on a stable regimen of standard pharmacological therapy for at least 3 months, with suboptimal clinical response. The intervention consisted of adding a food supplement containing natural extracts of *A. oleracea* and *B. serrata* to the ongoing therapy, at a dosage of two tablets per day. The follow-up period extended for 8 weeks after the introduction of the supplement. During this period, adjustments to the standard pharmacological treatment were permitted based on clinical judgment and individual patient needs.

### 4.7. Food Supplement

Nervana^®^ (Sanitas Farmaceutici, Italy) is a patented food supplement containing standardized *A. oleracea* and *B. serrata* extracts, formulated for enhanced bioavailability. In Italy, this combination is already marketed as food supplement in gastro-protected tablets to optimize its absorption. *B. serrata* is incorporated into a phytosome complex, leveraging a phospholipid-based delivery system to improve intestinal absorption. Meanwhile, *A. oleracea* is combined with beta-cyclodextrin, a cyclic oligosaccharide that facilitates rapid and efficient bioavailability, ensuring optimal therapeutic delivery.

Each daily dose of two tablets provides:

- *A. oleracea* (standardized to 3% alkamides): 120 mg (product code 4002000, Bernett SRL, Milan, Italy, affiliate of Indena SpA, (Milan, Italy).)
- *B. serrata* (standardized to 25% triterpenic acids): 120 mg ((product code 36BWP0090, Indena SpA, Milan, Italy).)

### 4.8. Outcome Measures

*Primary Endpoint:*

The reduction in neuropathic pain component, as defined by the change in PD-Q and NPSI scores at Week 4 compared to baseline.

*Secondary Endpoints:*

- Change in PD-Q score at Weeks 2, and 8.
- Change in NPSI score at Weeks 2, and 8.
- Change in NRS pain score at Weeks 2, 4, and 8.
- Improvement in quality of life (SF-12) at Weeks 2, 4, and 8.
- Reduction in concomitant analgesic use at Week 8.
- ODI assessment at Weeks 2, 4, and 8.

*Safety Assessment*:

All adverse events (AEs) and serious adverse events (SAEs) were documented and monitored throughout the study period. Events were classified based on spontaneous reporting by patients and structured questioning during scheduled visits. Investigators classified events according to severity and judged the likelihood of association with the supplement on severity and potential causality to the investigational product.

### 4.9. Statistical Analysis

The primary analysis compared PD-Q scores at Week 4 with baseline using the Wilcoxon signed-rank test (two-tailed) at a significance level of 0.05. Secondary analyses evaluated changes in NRS, NPSI, ODI, SF-12, and medication use using repeated-measures ANOVA or non-parametric tests as appropriate. For multiple comparisons across time points, we applied Tukey’s multiple comparisons test to control for Type I error inflation, with adjusted *p*-values reported in the results. Potential confounding factors, including age, gender, BMI, pain duration, and baseline medication use, were assessed for their relationship with primary outcomes using correlation analyses and included as covariates in sensitivity analyses of the primary outcome.

Descriptive analysis was performed for safety outcomes.

No multivariate regression was performed, as this was an exploratory observational cohort aimed primarily at describing symptom evolution rather than estimating causal effect.

All statistical analyses were performed using GraphPad Prism version 10 (GraphPad Software, San Diego, CA, USA).

## 5. Conclusions

Within the inherent limitations of an observational design, this study offers preliminary evidence suggesting that a combined extract *of A. oleracea* and *B. serrata*, when added to ongoing standard therapy, may contribute to pain relief and functional improvement in patients with CLBP exhibiting neuropathic characteristics. The observed reductions in pain intensity and improvements in quality of life and functional measures indicate that the extract could serve as a supportive adjunct in multimodal pain management strategies.

While the exact contribution of the supplement cannot be isolated due to the absence of a control group, the consistent trends across multiple validated measures and the favorable tolerability profile are encouraging. The extract was generally well tolerated, with only modest and transient side effects reported, and no serious adverse events.

These findings are consistent with preclinical data suggesting potential synergistic actions of the included botanicals on inflammatory and neuropathic pain pathways. However, further randomized, placebo-controlled studies with longer follow-up periods are necessary to confirm these results, better define the clinical utility of the extract, and explore its possible role as a standalone option in patients who cannot tolerate conventional analgesics.

## Figures and Tables

**Table 1:** Baseline demographic and clinical characteristics of the study population. BMI: body mass index.

**Total Patients Enrolled**	103
**Mean Age (±SD)**	63.1 (±14.3) years
**Mean BMI (±SD)**	26.4 (±4.2) kg/m^2^
**Gender Distribution**	Female: 57 (55.3%) Male: 46 (44.7%)
**Comorbidities:**	
- Cardiovascular disease	24 patients (23.3%)
- Diabetes Mellitus	8 patients (7.8%)
- Osteoporosis	7 patients (6.8%)
- Chronic obstructive pulmonary disease (COPD)	5 patients (4.9%)
- Hypercholesteremia	5 patients (4.9%)
- Rheumatological disease	5 patients (4.9%)

**Table 2:** Baseline pharmacological treatments received by patients enrolled in the study.

Drug Class	Number of Patients
**NSAIDs** (Ibuprofen, Ketoprofen, Diclofenac, Nimesulide)	39
**Opioids** (Tapentadol, Buprenorphine, Tramadol, Oxycodone/Naloxone, Fentanyl)	30
**Gabapentinoids**	27
**Paracetamol**	22
**Muscle Relaxants** (Thiocolchicoside, Tizanidine)	5
**Cannabis-based Medications**	2
**No Treatment**	8

**Table 3:** Changes in Neuropathic Pain Symptom Inventory (NPSI) subscores from baseline to Week 8. n.s. indicates not statistically significant compared to baseline.

Outcome	Week 2 (*p* Values Compared to Baseline)	Week 4 (*p* Values Compared to Baseline)	Week 8 (*p* Values Compared to Baseline)
Total pain score	*p* < 0.0001	*p* < 0.0001	*p* < 0.0001
Burning sensation	n.s.	n.s.	*p* < 0.05
Pressure sensation	n.s.	*p* < 0.05	*p* < 0.0001
Squeezing sensation	*p* < 0.05	*p* < 0.01	*p* < 0.0001
Electric shock	*p* < 0.05	*p* < 0.0001	*p* < 0.0001
Evoked by brushing	*p* < 0.001	*p* < 0.0001	*p* < 0.001
Stabbing	n.s.	n.s.	n.s.
Evoked by pressure	n.s.	n.s.	*p* < 0.0001
Evoked by cold stimuli	n.s.	n.s.	n.s.
Pins and needles	*p* < 0.05	*p* < 0.05	*p* < 0.0001
Tingling	*p* < 0.0001	*p* < 0.0001	*p* < 0.0001

**Table 4:** Change in concomitant medication use over the 8-week study period.

Medication Class	Baseline	Week 2	Week 4	Week 8	% Reduction	*p* Values
NSAIDs on demand	39	39	37	30	23.07	*p* < 0.05
Opioids	30	29	29	28	6.66	n.s.
Neuromodulators	27	26	24	21	22.2	*p* < 0.05
Paracetamol	22	22	22	22	0	n.s.
Muscle Relaxants	5	4	4	4	20	n.s.
Cannabis	2	2	2	2	0	n.s.
No treatment	8	8	9	12	−50.00	n.s.

**Table 5:** Summary of adverse events observed during the 8-week study period.

Adverse Event Category	Number of Patients	Percentage of Patients (%)
Patients with adverse events	9	8.7%
Gastrointestinal discomfort	4	3.9%
Transient dizziness	3	2.9%
Mild skin reactions	2	1.9%
Serious adverse events	0	0%
Discontinuation due to adverse events	0	0%

## Data Availability

The data presented in this study are not publicly available due to ethical and privacy restrictions but are available from the corresponding author upon reasonable request and pending approval by the relevant ethics committees.

## List of Abbreviations

AE	Adverse Event
AKBA	Acetyl Keto-beta-boswellic Acid
BMI	Body Mass Index
CHILD	Child–Pugh Classification
CLBP	Chronic Low Back Pain
COPD	Chronic Obstructive Pulmonary Disease
DN4	Douleur Neuropathique en 4 Questions
LBP	Low Back Pain
MCS	Mental Component Summary
NPSI	Neuropathic Pain Symptom Inventory
NRS	Numeric Rating Scale
NSAIDs	Non-Steroidal Anti-Inflammatory Drugs
ODI	Oswestry Disability Index
PCS	Physical Component Summary
PD-Q	painDETECT Questionnaire
RCT	Randomized Controlled Trial
SAE	Serious Adverse Event
SF-12	12-Item Short Form Survey
TRP	Transient Receptor Potential
